# Effectiveness of m-Learning in Enhancing Knowledge Retention for Nurses’ Lifelong Learning: Quasi-Experimental Study

**DOI:** 10.2196/72957

**Published:** 2025-07-17

**Authors:** Daniel José Cunha, Paulo Machado, José Miguel Padilha

**Affiliations:** 1 Escola Superior de Enfermagem do Porto, R. Dr. António Bernardino de Almeida nº 830 - 844, 856, Porto, 4200-072, Portugal, 351 225073500; 2 RISE-Health, Porto, Portugal

**Keywords:** m-learning, nursing, continuing education, knowledge retention, COPD, MOOC, clinical virtual simulation, chronic obstructive pulmonary disease, Massive Open Online Courses, mobile learning

## Abstract

**Background:**

The current information and communication technologies, digital literacy, and ease of access to communication and information devices by nurses provide them with new ways and intention to access information for technical-scientific updating, ensuring the quality and safety of health care. Mobile learning (m-learning) offers a flexible and accessible alternative for continuing professional education, overcoming barriers such as time constraints and financial burden.

**Objective:**

This study aimed to evaluate the effectiveness of m-learning in nurses’ knowledge retention of chronic obstructive pulmonary disease self-management, using a Massive Open Online Course with integrated virtual clinical simulation.

**Methods:**

A quasi-experimental pre- and posttest study was conducted, with no control group, among 168 nurses from a Portuguese hospital. The intervention included an asynchronous online course with 13 modules. Knowledge retention was assessed by comparing the mean scores before and after the course.

**Results:**

The results indicated a significant increase in knowledge retention. The participants’ average score increased from 59.97% in the initial assessment to 84.05% in the final assessment (*P*<.001). Nurses with a master’s degree exhibited a higher level of basic knowledge than those with a bachelor’s degree. The course completion rate was 93.45%, reflecting significant engagement attributed to gamification and clinically relevant content.

**Conclusions:**

This study confirms the effectiveness of m-learning in improving knowledge retention in nursing. This strategy is a valuable approach to lifelong learning, promoting quality and safety in delivering health care. m-learning is useful in nurses’ lifelong learning, offering flexibility and more effective support for clinical practice. Integrating virtual simulation and gamification boosted motivation and reduced drop-out rates, highlighting the potential of m-learning in lifelong learning in health care.

## Introduction

### Background

Available information and communication technologies (ICT), digital literacy, and easy access to communication and information devices provide nurses with new opportunities for technical and scientific updating. These advancements are directly linked to the safety and quality of care [1]. However, nowadays, there are still barriers to accessing lifelong learning [2], such as the need to balance family and professional life and the associated financial burden [3,4]. On the other hand, the decrease in human resources and their turnover and the increase in workload in health institutions are obstacles to nurses’ continuing training [5,6].

The use of ICT-based platforms by health care institutions as a strategy for providing lifelong learning to nurses enables large-scale access with no geographical and time limits [7-9].

The World Health Organization defines a digital health intervention as the specific use of digital technology to achieve health-related objectives. An example of these interventions is managing training and educational content and making it available to health professionals in digital format [10]. Digital education includes computer-based offline and online education, open and massive online courses, serious games and gamification, augmented reality environments, virtual reality, virtual simulations of clinical cases, psychomotor skills training, and mobile learning (m-learning), among others [11].

e-learning, electronic learning or web-based learning often complement traditional teaching models, encompassing knowledge through digital technologies. These educational approaches promote access to training for health professionals [10,12].

The technological development has leveraged changes in education and fostered the use of mobile technologies in the acquisition of knowledge and its mobilization for clinical practice [13]. Current distance education contexts demand the use of mobile devices in addition to traditional computers [14].

We have been witnessing the progressive introduction and incorporation of e-learning as a pedagogical strategy coupled with the growing access to portable distance communication devices, such as smartphones and mobile internet networks. Within this new reality, m-learning has emerged as a new opportunity to promote nurses’ access to digital education as a critical resource for lifelong learning. m-learning is the management and provision of education and training content in electronic format for health care professionals [10]. It allows users to access educational content via mobile devices, regardless of time and location [15]. Evidence shows that m-learning is at least as effective as traditional learning [11].

Massive Open Online Courses (MOOCs), made available by higher education institutions [16], certified and integrated into the in-service training programs of health institutions, are emerging as important contributors to incorporating m-learning in the continuing training of health professionals. Moreover, MOOCs can contribute to developing nurses’ skills, specifically in training for self-management of noncommunicable chronic diseases such as chronic obstructive pulmonary disease (COPD).

### Massive Open Online Courses

MOOCs are based on the definition of various concepts such as e-learning, mass communication, knowledge sharing, and openness [17]. MOOCs comprise quality content and aim at a large target audience, overcoming many of the in-class barriers [9]. With over 20 years of existence, these courses stand out for their creative methodology for conveying information and implementing innovative pedagogy and tools [18]. The MOOCs’ primary goal in the health field is to improve the health status of the general population while promoting the safety and quality of the care provided [8].

In recent years, the number of MOOCs has grown exponentially, in line with the increase in the number of people with access to the internet [19,20]. In 2021, around 220 million people enrolled in online courses, representing twice the number of students enrolled in 2020 [21].

This reality explains the upward trend in using MOOCs, as they are easy to access and tend to be free [22]. The size of the global MOOC market was US $9.45 billion in 2023, estimated to reach US $39.72 billion by 2032 [23].

MOOCs comprise several characteristics, such as openness, which means the possibility of enrolling and withdrawing from the course; autonomy, referring to the pace of learning and goal attainment; diversity, which allows adaption to different time zones; and interactivity, where the effects of learning picture the sharing between participants [8].

MOOCs have been perceived as an innovative method enabling useful and effective online learning compared to face-to-face training [8]. Its success relies on the number of trainees that can be enrolled swiftly; its ability to respond to individual learning styles by using multiple tools for interaction between participants; its flexibility [2] and the guarantee of quality learning associated with cost savings in professional training.

In nursing education, the use of MOOCs has fostered the creation of broader learning opportunities for individuals and health institutions [24].

A MOOC must be designed considering the needs of the participants [25] and increase motivation and engagement. Continuous training that aims to respond to nurses’ needs and is perceived as positive for the quality and safety of the care provided is more attractive and likely to better capture nurses’ intention to attend and complete the course [26].

MOOCs used to develop professional competencies show higher enrollment and completion rates compared to more generic content [27,28].

The number of drop-outs in trainees from MOOCs before completion has been under analysis. In a study with 9 million students, only 5% to 18% of participants completed the course [29]. Another study reported a MOOC completion rate of less than 10% [30]. More recently, MOOC drop-out rates have ranged from 10% [31] to 85% [32].

The advantages and personal and professional gains for individuals who attend MOOCs are irrefutable, considering the impact on learning and overall knowledge pertaining to the development of skills, attitudes, confidence, and commitment [33].

Incorporating gamification features into MOOCs and using virtual patients for clinical decision training could help increase the involvement and motivation of participants to complete MOOCs [6,17,34]. MOOCs should integrate virtual patient simulators, recreating gamified environments, which are predictors of realism, fun, and motivation for participants, and reduce the drop-out rate [4,35], thus increasing MOOC completion rates [36,37].

However, little is known about the effectiveness of using m-learning for nurses’ lifelong learning. Therefore, this study sought to assess the effectiveness of an m-learning strategy on nurses’ knowledge retention.

## Methods

### Study setting

A quasi-experimental pre- and posttest study was conducted without a control group, using a nonprobabilistic convenience sampling technique. All nurses from the Internal Medicine Department of a central hospital in northern Portugal who voluntarily agreed to participate in the study were considered eligible. Thus, all 193 nurses from the Internal Medicine departments were eligible to participate.

The primary outcome of the study was the nurses’ level of knowledge, analyzed using a continuous variable expressed as a percent (0‐100). The calculation of the average level of knowledge before and after attending the MOOC resulted from computing the relative weight of each module included in the intervention. Knowledge retention was calculated by testing the differences between the average percent obtained before and after the intervention.

### Intervention

The development of the MOOC was supported by the theme of self-management of COPD, considering the need expressed by the professionals within the study context and according to the Global Initiative for Chronic Obstructive Lung Disease (GOLD). The GOLD initiative reveals that COPD is now one of the 3 leading causes of death in the world, and 90% of these deaths occur in low- and middle-income countries. Many people suffer from this disease for years and die prematurely because of it or disease-associated complications. Globally, the burden of COPD is expected to increase in the coming decades due to continued exposure to COPD risk factors and an aging population [38].

This study used the Adult Learning Theory of Malcolm Knowles (1913‐1997), since for effective learning, adults—nurses in this case—have to perceive the usefulness of the knowledge provided and how to mobilize it for their daily practice [39].

This study included a training program available in MOOC format, called self-management of COPD (Ecare-COPD). This program has been subject to continuous validation, with special highlight for its adequacy and scientific and pedagogical relevance [6]. This MOOC was made available on NAU, an e-learning platform, created and administered by the Scientific Computing Unit (FCCN) of the Foundation for Science and Technology (FCT). This model supports training and enables the design of training programs in Portugal using the MOOC format.

This MOOC was organized into 13 modules, 12 of which are formative and include lessons on a topic. Each lesson includes a theoretical overview of the topic, one or more supporting videos (lasting about 5.06 min) and bibliographical references.

In addition, in module 13, which was not assessed, each participant was able to practice clinical decision-making through four clinical scenarios using the Body Interact virtual patient simulator [35,40-43]. In this module, each participant had the opportunity to mobilize the previously acquired knowledge using the game methodology.

### Data Collection

Sociodemographic variables and variables characterizing the professional category of the participants were used to characterize the sample.

The level of knowledge was assessed using a set of items, including 29 multiple-choice questions and 24 true or false statements, on the clinical area of COPD. The assessment items were determined by the experts who developed the contents of the training program and validated by a group of experts in the field of the training program.

It should be noted that the evaluation questions were pretested with 10 nurses. At this stage, only editing corrections were deemed necessary and no question was amended. Knowledge assessment was performed through an initial knowledge assessment of the participants after registering on the NAU platform. All participants were able to attend the training program despite the initial assessment. Each participant was asked to complete a knowledge assessment at the end of each module.

### Data Analysis and Processing

The statistical analysis was performed using SPSS Amos (version 29.0; IBM Corp) and SPSS (version 29.0; IBM Corp) software. Descriptive statistics and inferential analysis were used to analyze the different variables under study. The Student *t* test was used to test for differences between means. The results are reported according to APA standards, with Cohen *d* effect magnitude (0.2 low; 0.5 mean and 0.8 high) and *P* values set at <.05.

### Ethical Considerations

Ethical approval was granted by the Ethics Committee of CHUPorto/ICBAS (reference number 2021.010). All participants received an informed consent form and the anonymity and confidentiality of the data collected was guaranteed. We have also ensured that participants have the option to participate and will not suffer any damage or loss in case they do not complete the training program.

## Results

This study included 168 nurses from the medicine department of a hospital in northern Portugal, representing 87% (n=193) of the total nursing staff in the department. The remaining 13% (n=25) of potential participants were absent due to temporary work incapacity, extended health-related leave, and parental leave.

Of the participants, 69.6% (n=117) were female, 11.3% (n=19) were male, and 19% (n=32) preferred not to answer. The mean age of nurses was 32.96 (SD 9.37; 24-59) years; average professional experience as a nurse of 10.12 (SD 7.50; 0-37) years, and an average experience of 6.43 (SD 6.22; 0-26) years in the Medicine Department of this hospital in the north of Portugal. Further, male nurses were older (*t*_131_=−2.674; *P*=.004; Cohen *d*=0.67; men=37.94 and women=32.70), had longer professional experience (*t*_132_=−3.401; *P*<.001; Cohen *d*=0.86; men=15.17 vs women=9.26), and had longer experience in the medical department (*t*_131_=−2.615; *P*<.005; Cohen *d*=0.66; men=10.17 vs women=6.11). In this study, 74.4% (n=125) of the nurses held a bachelor’s degree in nursing, 11.9% (n=20) held a master’s degree, and 13.7% (n=23) opted not to disclose this information. Regarding professional categories, 66.1% (n=111) were general nurses, 19.6% (n=33) were specialist nurses, 1.2% (n=2) were nurse managers, and 13.1% (n=22) did not provide any information.

Regarding attendance at the training program, data analysis revealed that out of 168 participants, 157 successfully achieved an evaluation score exceeding 50%, thereby qualifying for a certificate of participation and completion. Among these, 99.36% (n=156) were issued certificates, while 0.63% (n=1) had still not received their certificates at the time of data extraction. In addition, 6.54% (n=11) of enrolled participants did not complete the training program despite completing their registration.

The participants completed an initial knowledge assessment, achieving an average score of 59.97% (SD 19.37; 0-97). The final knowledge retention assessment was calculated by determining the relative weight of each module. Participants scored on average 84.05% (SD 24.29%; 0-100) in the final assessment.

Descriptive statistics for the evaluation of the 12 modules included in the training program are described in Table 1.

**Table 1.** Descriptive data for the assessment of the 12 modules of the formative program.

Modules assessed	Participants (N=168), mean (SD; range)
Module 1	92.01 (17.81; 33-100)
Module 2	80.70 (27.49; 0-100)
Module 3	98.11 (8.58; 33-100)
Module 4	88.28 (14.33; 40-100)
Module 5	92.48 (14.04; 40-100)
Module 6	83.33 (24.97; 0-100)
Module 7	88.85 (16.60; 25-100)
Module 8	58.97 (49.35; 0-100)
Module 9	67.95 (46.82; 0-100)
Module 10	83.44 (37.29; 0-100)
Module 11	91.28 (16.09; 20-100)
Module 12	87.90 (32.72; 0-100)

Statistically significant differences in knowledge retention were found between the mean initial assessment score of 59.97 (SD 19.37) and the final assessment score of 84.05 (SD 24.29; *t*_167_=16.697; *P*<.001), with a large effect size (Cohen *d*=1.28).

An analysis of the relationships between sociodemographic and professional characteristics revealed a statistically significant difference in knowledge retention between the mean initial assessment scores of 69.15 (SD 12.77) among nurses with a master’s degree, while those with a bachelor’s degree had a mean score of 62.52 (SD 13.62; *t*_143_=−2.037, *P*=.02, Cohen *d*=0.49). These findings demonstrate that nurses with a master’s degree have a higher baseline knowledge on this subject compared to those nurses with a bachelor’s degree.

These study results confirm that the implemented pedagogical strategy significantly enhances knowledge retention among nurses within this specific context.

## Discussion

### Principal Findings

This study included a sample of 168 nurses from the medicine department of a hospital in northern Portugal who achieved an average score of 59.97% on the initial knowledge assessment. Throughout the training program, participants underwent final knowledge assessments at the end of each module, resulting in an overall average score of 84.05%. These findings demonstrate that the pedagogical strategy using ICT delivered through a MOOC and incorporating virtual clinical simulation significantly enhanced knowledge retention among nurses (*P*<.001, Cohen *d*=1.28).

Numerous studies on educational programs support these findings, highlighting their effectiveness in improving knowledge outcomes [44-47].

The positive impact of interventions involving simulation on knowledge acquisition is also well documented, although primarily in studies with nursing students. These findings underscore the value of active learning strategies [48], which play a critical role in motivating trainees to acquire knowledge [49,50]. This evidence reinforces the rationale for integrating virtual clinical simulation and virtual patients into the MOOC’s content.

This study also revealed that nurses holding a master’s degree exhibited a higher level of knowledge in the initial assessment of this area compared to those with a bachelor’s degree. These findings suggest that the second study cycle facilitates the consolidation of cognitive and instrumental skills and equips nurses with the ability to manage complex situations, such as empowering patients to self-manage chronic conditions such as COPD. Consequently, a master’s degree is expected to enable nurses to deliver specialized and targeted interventions that address the actual needs of the population, implement evidence-based complex interventions, and promote the quality and safety of care.

The training program was conducted in a MOOC format and incorporated multimedia resources (eg, videos) and brief summaries of the latest evidence on COPD. These features enhance the perceived usefulness of a MOOC [51], fostering greater engagement and commitment to the learning process [52] while increasing participants’ intention to enroll in similar courses in the future [53].

These factors may help to explain the low drop-out rate observed in this training program (6.55%), which is notably lower than those reported in previous studies [6,29-32,54].

As previously mentioned, the alignment of the MOOC with the participants’ professional roles [27,28], specifically its focus on developing competencies to empower individuals with COPD to improve self-management of the disease, likely contributed to higher enrollment and completion rates.

In addition, the flexibility provided to participants to complete the training program [55] may have further supported the high completion rate by accommodating the professionals’ needs to balance personal, family, and work responsibilities.

The use of simulation is increasingly significant in pre- and postgraduate programs [56]. High-fidelity simulation enables recreating a situation or event in which a virtual patient represents or responds to physiological parameters, providing high realism and dynamism for those involved [57]. This helps to optimize engagement in the learning process. High-fidelity simulation, which can include simulation with virtual patients, improves learning satisfaction, confidence, and self-efficacy among nursing students [58-60].

Virtual patient simulation has been used effectively to improve learning, specifically knowledge, self-confidence, practical skills, student satisfaction, and critical thinking [35,61]. It contributes to transforming learning environments into more interactive and creative scenarios, facilitating participants’ learning and achieving goals [62]. The integration of simulation with virtual patients, along with the gamification component of the training program, played a pivotal role in promoting adherence and achieving higher completion rates. Using simulation with virtual patients is an effective strategy for fostering active learning and stimulating intrinsic motivation to learn. This approach enabled the recreation of clinical decision-making environments and scenarios that participants were familiar with, allowing them to identify opportunities to enhance their decision-making skills when addressing daily challenges, in line with the findings from previous studies [4].

### Limitations

This study had some limitations. The first limitation concerns the study’s methodology, mainly because of the absence of a control group. This prevented attributing knowledge gains to the developed intervention. Further randomized controlled studies are suggested to confirm the results.

Also, since the sample was restricted to one medicine department in a single hospital institution, it hindered the generalizability of the results. Future research should include samples from different healthcare contexts.

In addition, the study was limited only to one intervention related to COPD self-management; thus, further investigation including other areas and dimensions of nursing practice is needed to examine the impact of implementing a pedagogical strategy in the continuous training of nurses.

In addition, future studies should focus on the longitudinal monitoring of the sample, particularly to assess knowledge retention on the subject under study.

### Conclusions

The adopted training program improved knowledge retention. Therefore, it is an alternative for optimizing nurses’ lifelong learning according to the program features, which have highly contributed to the completion rate (93.45%).

The obtained results provide relevant information to health institutions and their managers concerning the characteristics of a pedagogical strategy for the continuous training of nurses. These results align with the professionals’ needs and attest to the quality and safety of care.

Similarly, the results of this study should enable higher education institutions responsible for designing MOOCs to reflect on their content and reformulate or improve them.

## Abbreviations

COPD: chronic obstructive pulmonary disease
GOLD: Global Initiative for Chronic Obstructive Lung Disease
ICT: information and communication technologies
m-learning: mobile learning
MOOC: Massive Open Online Course
WHO: World Health Organization
